# The Role of Neuroinflammation in the Pathogenesis of Hepatic Encephalopathy

**DOI:** 10.1155/jimr/6855563

**Published:** 2025-10-25

**Authors:** Kathryn Rhodes, Yubo Wang, Sharon DeMorrow, Poorani Gurumallesh

**Affiliations:** ^1^ Pharmacology and Toxicology Division, College of Pharmacy, University of Texas at Austin, Austin, Texas, USA, utexas.edu

**Keywords:** astroglia, choroid plexus, microglia, neuroinflammation, proinflammatory cytokines

## Abstract

Liver disease impacts millions of Americans every year, which is compounded by the comorbidities and consequences that patients are susceptible to developing. Hepatic encephalopathy (HE) is a severe consequence of liver failure resulting in a range of cognitive deficits that heavily impact quality of life. Approximately 40% of acute liver failure (ALF) patients and 50% of chronic liver disease patients will be diagnosed with HE, and the associated prognosis is 44% and 42%, respectively. Though understanding of some of the neurologic impacts of liver impairment exists, the pathology of HE is not yet fully elucidated. Many in the field have come to appreciate the role of neuroinflammation in its pathogenesis. In this review, we have summarized recent studies investigating aspects of neuroinflammation such as microgliosis, astrogliosis, proinflammatory cytokine and chemokine production, and the involvement of the choroid plexus and meninges in HE.

## 1. Introduction

Liver disease, both acute and chronic, affects up to 100 million Americans, resulting in widespread complications to other organs [1]. Patients with liver disease can experience comorbidities such as hepatic encephalopathy (HE), which describes a spectrum of motor and cognitive deficits. Up to 40% of patients with acute liver failure (ALF) and 51% of those with chronic liver disease will develop HE [2, 3]. As of 2020, the incidence of HE in cirrhosis patients has increased from 7% to 42% [4], showing how diagnostic sensitivity has increased since the incidence of cirrhosis has only increased from 0.27% in 2015 [5] to 2.6% in 2022 [6]. Due to the complex nature of the disease, HE patients require assistance from both caregivers and health care systems to aid in treatment, presenting a financial burden on both. Total HE in‐patient charges increased from $8.15 billion in 2010 to $11.9 billion in 2014 [7], with costs estimated to increase coinciding with the increased incidence rate. Based on these statistics, it is evident that HE is a growing prognostic concern for patients with impaired liver function and creates a significant burden on the health care systems available for their treatment.

The physical manifestation of the neurologic symptoms that can be observed in patients of acute and chronic liver disease‐induced HE depend on the severity of the HE event and include personality changes, erratic behavior, hallucinations, altered sleep–wake cycles, and deficits in learning and memory ability [8, 9]. Patients can experience more motor deficits like ataxia, trouble maintaining balance, and impaired locomotion [8, 9]. These symptoms and their severity depend on the grading scale for HE, where cases are described from covert, or minimal, to overt, or severe, HE.

Covert HE, also referred to as minimal HE, typically describes the early stages of chronic HE where the beginnings of mild cognitive deficits start to show [2]. Because the physical manifestations of the deficits are so subtle, most diagnostics at this stage require biomarker evaluation, such as blood ammonia levels, in addition to specialized psychometric tests such as the Stroop test and the critical flicker frequency test to indicate minor disturbances to cognitive ability [2]. Upon these personality and memory deficits becoming gradually more apparent to those unfamiliar with the patient’s normal state, the HE can be deemed as overt [2]. Severe, or overt, HE is characterized by disorganized or uncoordinated motor movements, marked confusion, and altered speech patterns alongside the personality changes and other deficits similarly observed in cases of minimal HE [2]. In the most severe cases, overt HE can result in coma, due to cerebral edema, and death [7]. Many of these symptoms do not resolve after resolution of the liver disease, suggesting permanent, long‐term alteration in brain function can occur [10]. Furthermore, chronic HE patients may experience episodic HE, which is the disappearance and reappearance of some or all these cognitive symptoms that may worsen with each episode [8].

Prognostically, 44% of acute and 42% of chronic liver failure patients succumb to the illness within 12 months of HE diagnosis [2, 11]. The poor prognosis rates associated with HE may be attributed to the absence of targeted therapeutics for the treatment of HE. The current standard of care focuses on liver transplantation, management of patient diet and lifestyle, and treating the hyperammonemia with pharmaceutics such as Xifaxan (rifaximin) and lactulose, among others. However, there are no treatments for preventing or blunting the cognitive impacts of either type of HE, indicating a heightened need for further research into the mechanisms by which these impacts are sustained.

**Figure 1:**
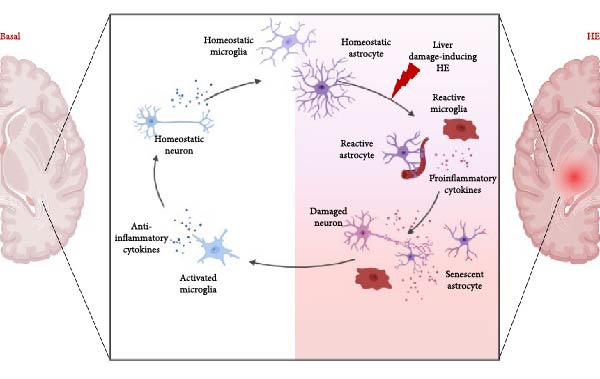
Pro‐inflammatory progression of microglia and astrocytes from basal state during pathogenesis of hepatic encephalopathy. A fine balance between pro‐ and anti‐inflammatory pathways exists to regulate the brain’s status from basal to disease state. In hepatic encephalopathy, many factors cause the activation of microglia and astrocytes in many brain regions as a result of liver injury that precipitates into hepatic encephalopathy. Upon the activation of these cell types, secondary effects take place that can contribute toward behavioral and cognitive phenotypes associated with hepatic encephalopathy, such as astrocyte senescence and microglial phagocytic activity.

In this review, we will discuss the most current findings of multiple aspects of neuroinflammation in the pathogenesis and progression of both acute and chronic HE. Specifically, the contribution of microglia, astrocytes, proinflammatory cytokine and chemokine signaling, and the involvement of the choroid plexus and meninges in HE pathology (Figure 1).

## 2. Microglia

**Figure 2:**
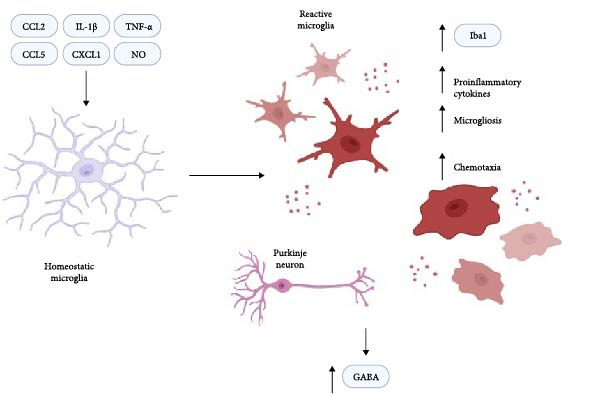
Roles of microglia in hepatic encephalopathy. When microglia at homeostasis receive a proinflammatory signal during HE pathogenesis, they become reactive, and their morphology reflects this by taking on an ameboid appearance and retracting their branches. These now reactive microglia express higher levels of Iba1, release their own pro‐inflammatory cytokines, encourage the recruitment of other microglia, and will move toward areas indicated as damaged during the disease. In addition, the pro‐inflammatory cytokines secreted by activated microglia may play a role in the increased release of GABA by Purkinje neurons to contribute to cognitive deficits.

Microglia are the innate immune cells of the brain and act as a crucial regulator of neuroinflammation in response to damage or as pathogenic drivers in neurodegenerative and neuromodulatory diseases [12]. Upon activation through upstream signals, microglia become activated and will secrete proinflammatory cytokines and chemokines known to trigger downstream local immune cell recruitment [13]. Their activation alone allows them to perform phagocytosis of damaged local neurons and cellular debris [14]. Increasing evidence in HE pathology has shown microglia reactivity to be associated with characteristic cognitive and motor deficits. Cirrhotic patients diagnosed with HE display upregulated ionized calcium‐binding adaptor molecule 1 (IBA1), a microglia‐specific marker, in postmortem cortical tissue compared to cirrhosis patients without HE (Figure 2) [15]. Inflammatory markers known to activate microglia, such as C–C motif chemokine ligand 2 (CCL2), CCL5, C–X–C motif chemokine ligand 1 (CXCL1), and secondary messengers, including nitric oxide (NO), are also increased in postmortem human brain tissue in HE patients [15] and cultured rat microglia [16]. These associations have provided the foundation for investigations into the impact of microglia on responding and coordinating neuroinflammation during HE pathology (Figure 2).

### 2.1. Reactive Microgliosis

Microglia can become reactive in response to proinflammatory signals sent in part by neurons responding to damage during HE. Reactive microgliosis is known to occur through neuronal secretion of CCL2, interleukin‐1beta (IL‐1β), and tumor necrosis factor‐α (TNF‐α); with these signaling pathways creating a repeating loop as these same signals are sent by reactive microglia for transduction by astrocytes, neurons, and other macrophages [17–19]. When neurons have become severely damaged, upregulation of CCL2 will occur, resulting in increased transduction of C–C motif chemokine receptor 2 (CCR2) signaling in CCR2^+^ microglia to cause microgliosis and activation [17].

Recent studies investigating the role of reactive microgliosis through neuron‐derived proinflammatory chemokines have identified numerous chemokines of interest and have aided in further elucidating the role of neuroinflammation in HE pathology. Reactive microgliosis has been identified as a contributing factor in driving the progression of other neurologic disorders, such as epilepsy, as observed in a recent study investigating CCL2–CCR2‐mediated microgliosis [17]. Microgliosis triggered the activation of signal transducer and activator of transcription 3 (STAT3), which has been observed in both chronic illnesses and cancers of the periphery to promote proinflammatory responses that can result in degradation or phagocytosis of healthy, unaffected cells [17, 20]. Another study investigating reactive microgliosis in sepsis‐associated encephalopathy (SAE) vs. Type A HE, or HE onset from ALF, has shown that the morphology of these increased amounts of microglia varies between the two pathologies [21]. Both pathologies showed reactive microgliosis as a factor of neuroinflammation, but Type A HE microglia showed a less phagocytic morphology, and rather more hypertrophic phenotypes persisted [21]. In SAE, reactive microglia presented with gitter cell‐like phenotypes, which are associated with high phagocytic activity [21], indicating that Type A HE pathogenesis presents microglia with distinctive phenotypes to contribute toward pro‐inflammatory responses when compared to other forms of encephalopathy. This was further evidenced by another study investigating Type A HE in mice treated with acetaminophen to induce ALF. They showed mice with Type A HE had an increased population of microglia that favored proximity and contact with astrocytes, indicating that potential microglia‐on‐astrocyte or astrocyte‐on‐microglia signaling may be another step in the pathway of neuroinflammation [21].

Reactive microgliosis and its subsequent increased proinflammatory signaling have been implicated in contributing to altered neurotransmission that may be responsible for the phenotypic cognitive and motor disturbances. Purkinje neurons, located in the cerebellum, are highly GABAergic, involved in motor projections, and have been shown to secrete increased TNF‐α via nuclear factor‐kappa B (NF‐κB) activation during sustained hyperammonemia [22]. In this instance, the reactive microgliosis may be driven by Purkinje neuron‐derived TNF‐ α, but this signaling forms a feedback loop between microglia and neurons (Figure 2). Another recent study investigating TNF‐α in HE‐related neuroinflammation has revealed that both the tumor necrosis factor receptor 1 (TNFR1)‐CCL2‐BDNF‐tropomyosin receptor kinase B (TrkB)‐KCC2 and TNFR1‐NF‐kB‐glutaminase‐GAT3 pathways via elevated TNF‐α lead to increased GABAergic transmission by the Purkinje neurons and are associated with motor deficits induced by hyperammonemia [23], showing that microglia‐derived TNF‐α can drive the subsequent GABAergic signaling in the cerebellum. In rats with portacaval shunts (PCSs) to model HE resulting from portosystemic shunting, sildenafil was administered to increase cGMP as a means of regulating the GABAergic transmission being elevated during neuroinflammation in the disease state [24]. The motor coordination deficits were attenuated in sildenafil‐treated PCS mice as assessed by balance beam walking [24], emphasizing the association between the proposed role of TNF‐α in mediating neuronal transmission in relation to behavioral phenotypes observed during HE. These studies aid in further elucidating the role of neuron‐derived neuroinflammation through the recruitment and activation of microglia.

Other instances of microgliosis via neuron‐derived signaling persist in the literature surrounding HE and other neurologic disorders. A study utilizing a global nuclear factor E2‐related factor 2 (Nrf2) knockout mouse strain showed that reactive microgliosis and astrogliosis were increased because of ablated Nrf2, indicating Nrf2 may play a key role in regulating neuroinflammation and the recruitment of glial cells during amyotrophic lateral sclerosis (ALS). Nrf2, a transcription factor that regulates the homeostasis of cellular redox, is also involved in regulating the transcription of anti‐inflammatory and antioxidative pathways that are known to be negatively altered in neurodegenerative and neuroinflammatory disorders [25]. In the Nrf2 knockout mice, novel object recognition (NOR) testing revealed that an LPS injection induced increased learning and memory deficits [25]. While this study did not investigate HE specifically, Nrf2 has been implicated in regulating neuroinflammation in other neurodegenerative disorders such as Alzheimer’s disease, ALS, and both ischemic and hemorrhagic strokes [25], and thus, alterations to Nrf2 could also occur in HE to affect reactive microgliosis and astrogliosis. A recent study investigating the use of edaravone to decrease reactive oxygen species (ROS) and oxidative stress damage in the thioacetamide (TAA) model of Type A HE in mice resulted in improved motor ability via open field assessment and upregulated the expression of glutathione, superoxide dismutase, and glutathione reductase as a means of reducing oxidative stress [26]. Notably, Nrf2 mRNA and protein levels were also upregulated in total brain tissue with edaravone treatment, which was associated with decreased p‐NF‐Kb and iNOS [26]. While the study did not investigate the downstream effects of edaravone on microglial reactivity or morphology, they did record downregulated total brain expression of pro‐inflammatory cytokines, including IL‐6, IL‐1β, TNF‐α, and interferon‐gamma (IFN‐γ) [26], which indicates the likelihood of potentially attenuated microglial reactivity, but additional research would be warranted to confirm this. More work investigating if neuronal Nrf2 secretions are upregulated to impact glial cells during both ALF and chronic liver disease may allow for further understanding of HE neuroinflammatory processes.

### 2.2. Microglial Activation

Reactive microgliosis reflects the activation of microglia, as their recruitment requires a morphologic change to move toward the source of the signal to perform their phagocytic or myelination roles. Upon activation, microglial somas will swell up and expand while their processes thicken and retract inwards toward the cell body so they can travel to sites of damage. Earlier studies initially found that microglial activation was induced by blood–brain barrier (BBB) permeability and heightened influx of circulating neurotoxins that failed to be detoxified by the failing liver [27], but recent work over the last 10 years shows increasing evidence that microglial activation can be onset earlier than BBB changes in pathology.

Microglial activation in the cerebellum, hippocampus, and cortex has been observed during early steatotic liver disease, highlighting neuroinflammation and microglial function as potential early drivers of the neurologic condition [28]. Activated microglia have been documented to secrete proinflammatory factors, including IL‐1β and TNF‐α [28], which further drive neuroinflammation (Figure 2). As to the factors outside of those also required for reactive microgliosis, recent work is highlighting new signal transduction pathways potentially unique to HE that may lead to their activation. Nucleotide‐binding oligomerization domain‐like receptor protein 3 (NLRP3) inflammasomes in both in vitro and in vivo models of hyperammonemia have been found to be associated with the activation of microglia, indicating that mitochondrial ROS resulting from excess ammonia may be activating microglia in addition to neuron‐derived activation [29]. This microglial activation mediated by NLRP3 was also correlated with behavioral phenotypes consistent with HE [29]. In mice that underwent bile duct ligation (BDL) surgery to model Type C HE, locomotion was significantly decreased during the open field assessment, anxiety‐like behavior was significantly increased when assessed by elevated plus maze, and learning and memory ability assessed via a passive avoidance test was significantly decreased [29]; highlighting the association between neuroinflammation driven by microglial activation and the characteristic behavioral outcomes of Type C HE.

However, other work has highlighted that glutamine synthetase (GS) (astrocyte‐specific) knockout mice show decreased microglial activation, further indicating ammonia alone may not be sufficient to induce this [28]. CD47, likely stemming directly from the liver in response to severe damage and subsequent failure, has also been implicated in microglial activation [30]. CD47 knockout mice showed decreased hepatic cell death and attenuated microglial activation, resulting in significantly improved neurologic scoring and delaying the time to enter hepatic coma after azoxymethane (AOM) treatment as a means of inducing Type A HE, indicating a potential direct or indirect role of CD47 in driving neuroinflammation during HE in acute cases [30].

Microglia contribute to the cellular response to inflammation that may induce and sustain the early cognitive deficits seen in patients with HE. Further work regarding the role of microglia in HE pathology may require activation pathway‐specific knockouts in in vivo and in vitro models of Type A and Type C, or onset by chronic liver failure, HE, to identify which drivers of microgliosis and reactivity are most responsible for increased cognitive deficits.

## 3. Astrocyte Dysregulation

**Figure 3:**
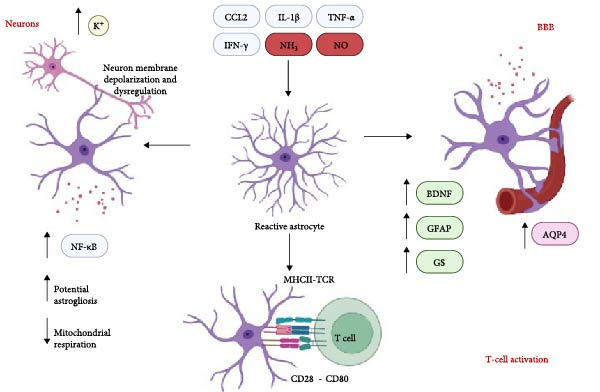
Roles of astrocytes in hepatic encephalopathy. Astrocytes become reactive during HE, resulting in many downstream pathways that may be responsible for the neurologic disturbances observed in patients. Astrocytes can affect neuronal membrane polarity, leading to dysregulation of neurotransmitter uptake and release. Astrocytes may express higher levels of NF‐kB due to their activation and may encourage the recruitment of other astrocytes. Astrocytes also experience metabolic changes in the form of decreased mitochondrial respiration, which may lead to their dysregulation and impact their ability to perform neuroprotective activities. At the blood–brain barrier (BBB), astrocytes interacting with ammonia and other neurotoxins experience changes in expression of enzymes and proteins responsible for neuroprotection, including brain‐derived neurotrophic factor (BDNF), glial fibrillary protein (GFAP), glutamine synthase (GS), and aquaporin 4 (AQP4). Astrocytes may also further drive neuroinflammatory responses during HE through MHCII‐TCR (T‐cell receptor) binding to T cells for activation.

Astrocytes are another glial cell of the central nervous system (CNS) that have roles, including supporting the structure of the BBB, modulation of neurotransmission and reuptake at synapses, detoxification of CNS toxins in the blood, supporting and regulating the growth of axons, and coordinating further neuroinflammatory responses to CNS damage [31]. Much like microglia, their activities and morphology will change in response to chemotactic signals like TNF‐α, IFN‐γ, and IL‐1β [32]. In the presence of IFN‐γ, astrocytes have been observed to express major histocompatibility complex (MHC) antigens, which can indicate their reactivity toward damage or pathogens present in the CNS [31, 32] (Figure 3). Similarly to microglia, astrocytes can also perform phagocytic activities under the correct stimuli to aid in an immune response toward harmful molecules [31]. In neurodegenerative diseases, traumatic brain injuries, and hyperammonemia, astrocyte somas have been observed to swell or enlarge, indicating reactivity in addition to upregulated GFAP expression [33, 34]. The processes of astrocytes have also been known to become polarized toward injury sites, and when lesions to the brain tissue have occurred, astrocytic processes will extend to aid in scar formation [33]. The role of astrocytes in detoxifying ammonia through glutamine synthase has been heavily studied in the context of hyperammonemia in HE, but recent studies are highlighting the role of astrocytes in the coordination of CNS neuroinflammatory responses as part of HE pathogenesis. Notably, astrocyte senescence has been observed in rat models of HE, and recent studies have begun to link this senescence to astrocyte inflammation and BBB permeability [35, 36].

### 3.1. Astrocytes and the BBB

The permeability and integrity of the BBB are highly important for HE patients, as ammonia is far from the only neurotoxin normally metabolized by the liver that may cross the BBB to incite further damage and initiate the onset of cognitive disturbances [37]. With astrocytes forming a critical layer of this sensitive tissue, their action or inaction is implicated in the pathogenesis of HE. Astrocyte endfeet are in contact with blood vessels, specifically through the tight junctions that form the endothelial cell layer. This allows astrocytes to aid in the regulation of the passive passage of molecules into the CNS across the BBB [31].

In a recent study, adult isolated astrocytes were cultured and exposed to 5 mM of ammonia (NH_4_Cl) for 24 h, which resulted in the upregulation of aquaporin 4 (AQP4) gene expression associated with the permeability of the BBB [37] (Figure 3). Additionally, elevated ammonia levels caused the cultured astrocytes to increase production and secretion of TNF‐α, BDNF, and IL‐6 into cell culture media, pointing toward astrocyte‐driven neuroinflammation in response to ammonia gliotoxicity commonly observed in HE [37] (Figure 3). This mechanism can be driven by the p38 mitogen‐activated protein kinase (MAPK) pathway, highlighted by the highly pro‐inflammatory response observed in the cultured astrocytes and an increase in AQP4 expression level [37]. The p38 MAPK pathway is related to pro‐inflammatory cascades associated with NF‐κB, as p38 MAPK can increase the transcription of NF‐κB to lead to downstream TNF‐α and IL‐6 production; correlating positively with the results observed in the study [33]. With respect to GS production, it was found that GS expression was upregulated in response to ammonia treatment, indicating a compensatory change for potential production against hyperammonemia [37] (Figure 3). These findings may indicate BBB permeability as one of the challenges astrocytes fail to overcome during HE, in addition to hyperammonemia alone.

Astrocytes in proximity to, and in contact with, the BBB may require more than just GS for ammonia‐induced swelling to occur, as observed in recent investigations. Na‐K‐2Cl cotransporter 1 (NKCC1) is an important ion transporter expressed by astrocytes that aids in fulfilling its role of regulating molecules that cross the BBB. Overactivation of NKCC1 in HE in models of Type A and Type C HE, and models of hyperammonemia, has been frequently observed, and inhibition of NKCC1 shows rescued astrocyte swelling [38–40]. This highlights the role of NKCC1 in facilitating astrocyte swelling and driving the resulting inflammatory response during HE. While the inhibition of NKCC1 may not be a viable therapeutic target for treatment, further work to elucidate the role of ion transporters in relation to BBB integrity can aid in further knowledge of HE pathogenesis.

### 3.2. Astrocytes and Ammonia

Astrocytes are incredibly helpful at coordinating inflammatory responses to protect the CNS, but in HE, they become dysregulated, which may cause them to drive pathology rather than serve a protective role. The metabolism of astrocytes in HE is known to be dysregulated via ammonia, altering intracellular pH levels to induce oxidative stress and producing ROS [36]. This results in mitochondrial dysfunction, senescence, and changes in inflammatory signaling and cascades driven by astrocytes affected [36]. This dysfunction spans further upstream from the electron transport chain into the TCA cycle, wherein glutamine and glutamate production are affected and thought to be partially responsible for some of the cognitive deficits in patients [36]. Additionally, these new pH levels from excess ammonia in the blood and within astrocytes attempting to metabolize ammonia through GS can drive the switch in glycolysis toward favoring lactate production [32]. This results in HE patients having decreased alanine levels; however, the opposite trend tends to be seen in animal and cell models [36]. One recent study heavily investigated this mitochondrial dysfunction and the impact of glutamate metabolism during HE found that human astrocytoma cultures with downregulated or inhibited the delta 2 glutamate receptor (GLUD2) (mitochondrial glutamate dehydrogenase) or glutamate dehydrogenase 2 (GDH2) with supplemented glutamine or glutamate aided in rescuing mitochondrial respiration [41]. They also showed through radiolabeled glutamine that hyperammonemia causes GHD2 to catalyze ammonia elimination via α‐ketoglutarate amination, leading to TCA cycle inhibition [41]. These findings may indicate a heightened role of GHD2 in the astrocytic response toward ammonia gliotoxicity.

Altered amino acid production, mitochondrial function, and metabolism in response to hyperammonemia are all avenues that require more work to identify how these alterations tie into driving neuroinflammation during HE pathology and how to make this coordination protective rather than degenerative. In models of Type A HE, astrocytes show downregulation of Kir4.1 expression at the mRNA level [42]. Kir 4.1 is another ion transporter responsible for inwardly rectifying potassium ions within astrocytes and is thought to be downregulated during states of high ammonia to decrease ammonia uptake to protect from swelling and gliotoxicity [42]. Downregulation of Kir 4.1 can result in increased extracellular potassium ions, which may induce neuronal depolarization and could be partially responsible for neuronal dysfunction driving decreased cognitive ability as assessed by the HE grading scale outlined by Zimmermann et al in 1989 [43] using both clinical and neurologic assessment of the stage of HE [42, 43]. This compensatory change shows that astrocytes can sense ammonia levels and do have ways to manage the toxicity to a certain degree, but more work investigating Kir 4.1 and other ion channels is needed to observe the fine balance between decreasing CNS ammonia content while preventing cerebral edema occurrence.

Another animal study investigating the transcriptional changes in glial cells using the BDL model showed that corticoid receptor and oxidative stress signaling were upregulated and that these changes occur over time (14–28 days postsurgery) [44]. The study also found that while chemotactic signaling was mainly upregulated in microglia, astrocytes displayed transcriptional changes related to a hypoxia response [44]. This included enrichment of *Hif3a*, *Gpx3*, *Ndrg1*, and *Txnip* via IPA analysis [44]. Corticoid receptor signaling via NR3C1 and NR3C2 receptors was identified to be associated with increasing further transcription of downstream corticoid response elements, including *Sgk1*, *Tsc22d3*, and *Fkbp5*; though these were seen to be most affected 14 days post‐BDL rather than the 28‐day time point [44]. Though this study specifically did not perform behavioral assessments to correlate these findings toward the cognitive deficits thought to be associated with astrocytes under hyperammonemic conditions, the BDL model alone has been well established as a means of replicating the cognitive and motor phenotype of chronic HE [29, 45]. These astrocyte transcriptional alterations identify compensatory, regulatory changes within astrocytes that may contribute toward their altered function and elucidate when their activity turns from beneficial to negative during HE (Figure 3).

### 3.3. Reactive Astrogliosis

Reactive astrogliosis, much like reactive microgliosis, is characterized by morphological, expression, and proliferation alterations in response to CNS injury or infection. Most notable is the increase in GFAP expression to indicate the intracellular changes the astrocyte makes to prepare to react. Morphological changes during this reactive state show enlarged cell bodies and, in some cases show decreased branch complexity as assessed by Sholl analysis. Astrocyte proliferation remains a controversial topic within the field of HE studies, as there is no clear consensus as to whether reactive astrogliosis, and subsequent GFAP upregulation, alongside astrocyte proliferation, occur in any type of HE reliably or in key brain regions rather than the brain in its entirety. Many believe that reactive astrogliosis may occur as a graded response toward the type of CNS insult sustained, in which only certain facets of the changes associated with reactive astrogliosis are observed (i.e., increased GFAP expression without increased astrocyte proliferation), and this may explain potential differences when comparing the phenotypes of astrocytes between the different HE pathologies.

Reactive astrogliosis is known to occur in other neurodegenerative and highly neuroinflammatory diseases such as Alzheimer’s disease [46]. In Alzheimer’s pathology, reactive astrogliosis is observed to be an early event that further progresses underlying etiology [46], and thus, the groundwork for the interest in astrogliosis as another key early event for HE pathology was laid. In strokes and trauma to the CNS, reactive astrogliosis can occur, resulting from a variety of factors known to also present in HE, such as disrupted BBB, oxidative stress, and pro‐inflammatory signaling [47]. Astrocytes also are shown to act in tandem as a population of cells working toward the same goal [47], rather than attempting to face damage as individual cells, lending more favor to the idea of reactive astrogliosis contributing to the overall neuroimmune response in HE.

In patients with hepatitis C viral (HCV) infections, which have been known to precipitate into HE stemming from the liver failure resulting from infection, reactive astrogliosis has been recorded and seemed to be correlated to the cognitive decline of the patients affected [48]. Even in patients without cirrhosis or diagnosed HE, reactive astrogliosis was observed in the basal ganglia and frontal and occipital white matter using magnetic resonance spectroscopy [48]. These changes for HCV‐specific liver injury show astrogliosis as an early response toward potential pathogen entry into the CNS, and this reactive astrogliosis has been observed in a more recent study investigating the neuropsychiatric symptoms of HE. Using the TAA mouse model of Type A HE, the study found that the cerebral cortex and the hippocampus showed higher levels of GFAP and astrocyte bodies 7 days after treatment [49]. This increase in astrocyte population was coupled with downregulated BDNF in the prefrontal cortex and downregulated IFN‐γ and IL‐6 in the hippocampus [49], potentially indicating the process of reactive astrogliosis and its downstream effects on coordinating neuroinflammatory responses during acute HE may be specific to each brain region. BDNF, a crucial neurotrophin, has a major role in coordinating the plasticity and development of GABAergic and glutamatergic synapses. BDNF can also modulate dopaminergic and serotonergic neurotransmission in brain regions associated with these projections. Changes in BDNF expression may contribute to the neuropsychiatric changes and cognitive disturbances in acute or chronic HE via these affected neuronal signaling pathways [50–52] (Figure 3).

It is evident that more work regarding the likelihood and role of reactive astrogliosis is required to determine the time and model‐specific nature of astrogliosis during HE pathology.

## 4. Proinflammatory Cytokines and Chemokines

Microglia and astrocytes both promote neuroinflammation by producing several pro‐inflammatory cytokines, such as interleukins IL‐1β and IL‐6, TNF‐α, chemokines like CCL2, CCL5, CXCL1, secondary messengers NO and prostaglandins, and ROS [53, 54]. These cytokines and chemokines can drive further cascades of pro‐inflammatory signaling that may positively or negatively affect one another’s functions or neuronal function during HE pathology.

### 4.1. Type A HE

In a study involving 16 patients with Type A HE caused mainly by acetaminophen hepatotoxicity, a significant correlation was observed between arterial cytokine levels of TNF‐α, IL‐1β, and IL‐6 and intracranial hypertension. Brain cytokine efflux was observed, aligning with cytokine production in the brain [55]. In an animal model of Type A HE, microglia have been demonstrated to be associated with the onset of HE and brain edema [56]. Without an inflammatory stimulus, microglia remain quiescent, actively engaging in surveillance. However, in response to inflammatory stimuli, these cells adopt an activated phenotype to prevent and manage CNS damage caused by disrupted homeostasis resulting from a wide range of insults from impending cerebral energy failure and metabolic lesions to cell death [57]. Type A HE, whether caused by hepatic devascularization in rats [56] or toxic liver injury in the mouse [58], leads to microglial activation and elevated brain levels of proinflammatory cytokines, including TNF‐α, IL‐1β, and IL‐6.

The expression of genes coding for TNF‐α, IL‐1β, and IL‐6 was found to be significantly increased, paralleling the rise in brain cytokine concentrations over time. Microglial activation and proinflammatory cytokine synthesis in the brain during Type A HE occur without neuronal cell death, indicating that neuroinflammation can arise from reversible cerebral metabolic disturbances rather than solely from neurodegeneration in Type A HE [59]. Mice undergoing the TAA model of Type A HE [60] show an increased brain weight and increased levels of TNF‐α, IL‐1β, and IL‐6. Treatment with the autotaxin inhibitor (ATXi) significantly ameliorated the neuroinflammation and reduced brain edema and the levels of IL‐1β, IL‐6, and TNF‐α [61]. Specifically, ATX inhibitor (HA130) significantly inhibited the levels of IL‐1β in serum, liver, and frontal cortex, IL‐6 in serum, and CCL3 in serum and frontal cortex from intervention groups compared to the TAA group [62]. Additionally, piracetam, a drug used clinically in neurological complications such as stroke and head trauma, significantly decreased brain levels of these inflammatory cytokines in TAA‐treated mice [63]. Salivarius Li01 significantly reduced TAA‐induced activation of the NF‐κB pathway, leading to decreased expression of IL‐6, CCL2, and chemokine CXCL1 in the serum [64].

In C57Bl/6 mice with AOM‐induced Type A HE [65], CCL2 is upregulated in neurons and binds to chemokine receptors on microglia. This leads to chemokine receptor‐mediated signaling, which results in the release of proinflammatory cytokines and subsequent neuroinflammation [66]. In certain pathologies, such as multiple sclerosis and cerebral ischemia, the main transmission mechanism of peripheral alterations is the infiltration of peripheral lymphocytes and/or monocytes into the brain [67–69]. The release of CCL2 attracts these immune cells to the brain. Elevated CCL2 expression in the hippocampus is linked to the recruitment of monocytes, microglia activation, neuronal dysfunction, and memory impairment [69–71]. Specific targeting of CCL2 activity through intraperitoneal injection of chemokine receptor 2 and chemokine receptor 4 inhibitors during ALF in AOM‐or TAA‐treated mice was found to reduce microglia activation and improve neurological function [72, 73]. The investigators also found that microglia have increased levels of CCL2, and intraperitoneal injection of anti‐TNFα serum led to reduced CCL2 expression in microglia [74]. Additionally observed in the AOM model was a downregulation of the anti‐inflammatory chemokine CX3CL1. Further, the injection of soluble CX3CL1 was found to reduce microglia activation and improve the time taken to reach coma, indicating that an imbalance of CCL2 and CX3CL1 expression may be driving the activation of microglia during Type A HE [75].

### 4.2. Type C HE and Other Chronic Liver Disease‐Derived Cytokine Observations

Patients with steatohepatitis often show mild cognitive impairment prior to the onset of cirrhosis [76, 77] and show neuroinflammation and neuronal loss in the hippocampus, along with increased IL‐1β and TNF‐α [78]. In cirrhotic patients with covert or minimal HE, plasma levels of many pro‐inflammatory cytokines, including IL‐6, IL‐21, IL‐17, IL‐10, IFN‐γ, IL‐18, CCL20, TNF‐α, CXCL13, IL‐15, and CX3CL1 are elevated, and TGF‐β is reduced. It is noteworthy that many of these pro‐inflammatory cytokines (IL‐17, CCL20, TNF‐α, CXCL13, IL‐15) promote the infiltration of cells from the peripheral immune system into the brain, which would induce neuroinflammation [79]. Type C HE induced by BDL and resection in both mice [74] and rats [80] results in microglial activation, which has been established with a range of cell‐selective markers. Interestingly, in bile duct‐ligated rats, microglial activation has been found to manifest brain regional selectivity. In rats with BDL, strong activation of microglia was demonstrated in the cerebellum, and mild microglial activation in the CA3 area of the hippocampus, corpus callosum, and piriform cortex, accompanied by the increasing inducible NO synthase [81], IL‐1β, and prostaglandin E2 (PGE2) [80]. In mice subjected to BDL, brain‐released CCL2 attracted infiltrating monocytes, causing neurological decline [74].

As for HE associated with indirect hepatic injury, end‐to‐side portocaval anastomosis in the rat led to elevated brain levels of the proinflammatory cytokine IL‐6, along with increased activities of cyclooxygenase (COX) and inducible NO synthase [82]. In a rat model of portal hypertension induced by triple calibrated portal vein ligation for 1 month, the CX3CL1 expression was not changed in the hippocampus or cerebellum, but its receptor CX3CR1 was significantly upregulated in both regions while stromal cell‐derived factor 1α and CXCR4 were upregulated in only the hippocampus [83]. In rats with portal vein ligation, Locomotor activity deficits were accompanied by increased expression of IL‐6 messenger RNA without any evidence of microglial activation [84].

### 4.3. Liver–Brain Axis and Systemic Inflammation

Evidence indicates that systemic proinflammatory mechanisms may trigger the liver–brain signaling process. The onset of systemic inflammatory response syndrome (SIRS) during Type A or Type C HE heralds a poor prognosis. As stated earlier, liver disease leads to liver damage and inflammation, hyperammonemia, and alterations in the gut microbiome. All these changes lead to peripheral inflammation and alterations in the immunophenotype and the content of extracellular vesicles in blood [85–87]. Brain signaling in SIRS potentially occurs via one of several mechanisms, including the active transport of cytokines across the BBB, interactions with receptors on circumventricular organs lacking the BBB, the activation of afferent neurons of the vagus nerve, infiltration of extracellular vesicles from the blood [23], or activation by peripheral pro‐inflammatory cytokines (IL‐6, IL‐17, TNFα) of their receptors in endothelial cells in the BBB and paracrine signaling to neighboring astrocytes in the brain [88–90].

It has been suggested that systemic inflammatory signals have the potential to result in increased permeability of the BBB to cytokines in those with acute or chronic HE. Ammonia is involved in the development of both Type A and Type C HE, including cytokines [91], and inflammatory cytokines like TNF‐α, IL‐6, and IL‐17 in the presence of hyperammonemia have been found to induce neurotoxicity by passing through the BBB and causing enlarged/swollen pale astrocytes, resulting in HE [92]. Ammonia is also believed to induce the production of pro‐inflammatory cytokines from brain microglia or endothelial cells in both pathologic states [93]. Extracellular vesicles from the plasma of rats with chronic HE and hyperammonemia elevate TNF‐α and its receptor TNFR1 in cerebellar slices from control rats, resulting in increased activation of TNFR1 [23].

In the cerebellum of rats with chronic hyperammonemia to model cirrhosis, increased BDNF levels promote TrkB activation in Purkinje neurons, triggering phosphatidylinositol‐3‐kinase (PI3K) activation. This process enhances the phosphorylation of AKT and of IkappaB (IκB), promoting the nuclear translocation of NF‐κB and enhancing TNF‐α content [22]. NF‐κB, as a main transcription factor, is activated in patients with acute and chronic HE and promotes swelling of astrocytes and brain edema, regulating inflammatory mediators such as TNF‐α, IL‐1β, IL‐6, and iNOS and apoptotic factors Bax and Bcl2, which are involved in neuroinflammation and apoptotic cell death in brain injury [22, 94–96]. Lactoferrin, an iron‐binding glycoprotein, modulated the rise in brain HMGB1/TLR‐4/MyD88/NF‐κB/TNF‐α signaling pathway through its anti‐inflammatory potential, guarding against TAA‐induced behavioral alterations [97]. Levetiracetam administration reduced the TAA‐induced expressions of IL‐1β, IL‐6, TNF‐α, and IFN‐γ by inhibiting the NF‐κB p65 activation in the brain. Microglia‐derived TNF‐α activates TNFR1 in Purkinje neurons, triggering the activation of NF‐kB and increasing IL‐17 and TNFα also in these cells. Enhanced TNFR1 activation also enhances activation of the TNFR1‐sphingosine‐1‐phosphate receptor 2 (S1PR2)‐CCL2‐BDNF‐TrkB pathway, which mediates microglia and astrocytes activation during hyperammonemia [98].

Anti‐TNF‐α, which is unable to cross the BBB, mitigates peripheral inflammation in rats by addressing chronic hyperammonemia‐induced neuroinflammation, neurotransmission changes, and cognitive deficits [99]. Moreover, in rats with hyperammonemia and minimal HE to model chronic HE, IL‐17 levels and membrane expression of the IL‐17 receptor are increased in the cerebellum, leading to increased activation of the IL‐17 receptor in microglia [99]. The activation of the IL‐17 receptor triggers activation of the STAT3 and NF‐kB, increasing IL‐17 and TNFα levels, respectively. GABA‐ergic tone in the motor cortex was also found to be increased in minimal HE (Grades 1 and 2) [100], and the GABA_A_ antagonist bicuculline decreases IL‐6 and TNFα and increases IL‐10 in the plasma of chronic hyperammonemia rats. It also reduces astrocyte activation, induces the microglia M2 phenotype, and reduces IL‐1β and TNFα in the cerebellum [101]. Golexanolone reduces peripheral inflammation and neuroinflammation and improves cognitive and motor function in hyperammonemia rats by reducing GABA_A_ receptor activation, which is related to the reversal of the hyperammonemia‐enhanced activation in cerebellum of the TNFR1‐glutaminase‐GAT3 and TNFR1‐CCL2‐TrkB‐KCC2 pathways [102]. While many of these studies highlight TNF‐α and its capacity as a potential target during neuroinflammation in chronic HE and chronic hyperammonemia, hyperammonemia is still a large factor in acute HE and thus warrants further studies to confirm if TNF‐α also exerts a similar role in relation to GABA‐ergic tone in Type A HE pathology.

### 4.4. Neuroinflammatory Therapeutics for HE

Therapies directly targeting neuroinflammatory processes focus on inhibiting microglial activation and the effects of proinflammatory cytokines it produces. Deletion of TNF‐α or IL‐1 receptor gene postpones the onset of encephalopathy. Similarly, the TNF‐α receptor antagonist etanercept decreases encephalopathy severity and prevents brain edema in ALF [58]. Minocycline, known for its strong inhibition of microglial activation independent of its antimicrobial effects [103], reduces proinflammatory cytokine production, slows the progression of encephalopathy, and attenuates brain edema in experimental ALF [104]. Intracerebral administration of JTE‐013 in hyperammonaemic rats can normalize the S1PR2‐CCL2‐CCR2‐BDNF‐TrkB‐KCC2 pathway by blocking S1PR2 and reducing glial activation and restoring motor coordination [105]. LNG exhibited a potent hepatoprotective activity and an effective neuroprotective potential that significantly hampered the progression of HE following TAA administration by modulating various inflammatory mediators: COX‐2, IL‐1β, and TNF‐α, together with the transcription factor, CCAAT/enhancer binding protein beta (C/EBPbeta), and the chemokine, CX3CL1, which modify microglial activation during neuroinflammation in acute HE [106].

Potential upstream potentiators of proinflammatory cytokine and neuroinflammation affecting synaptic plasticity may include the NO‐cGMP‐PKG cascade. Studies have found that NO‐cGMP can downregulate NF‐kB locally to decrease local inflammatory responses from interleukin secretion in chronic hyperammonemia [107]. Studies have also shown that administering a phosphodiesterase 5–6 inhibitor (PDE5I) in the TAA model of HE to inhibit the degradation of cGMP, and thus, increase brain concentrations, can attenuate reactive microgliosis and astrogliosis known to occur as a result of upregulated proinflammatory cytokine and chemokine production [107]. Preclinical trials for the use of PDE5I in neuroinflammatory disorders are underway and may prove to aid in combating some of the neurologic and memory‐based deficits in acute HE patients [107].

Pro‐inflammatory cytokines and their associations with the development and progression of cognitive deficits have been well documented in HE pathology, yet more work remains to be done with respect toward which cell populations in which key regions are most responsible in early pathology for inciting HE at the earliest stages.

## 5. Choroid Plexus and Meninges

In addition to the classic BBB, the choroid plexus and meninges have important roles in protecting the brain from peripheral insults and can modulate neuroinflammation [108, 109]. The choroid plexus is a small layer of tissue that aids in regulating the entry of components within the central ventricles, wherein the cerebrospinal fluid (CSF) is produced, into the rest of the CNS [110, 111]. It has been observed to swell in response to CNS insult or pathogenic exposure and will secrete inflammatory signals for phage activation [112]. During HE, patients and rodent models display increased levels of bile acids and ammonia in the blood and CSF from the damaged liver, leading to the idea that there may be a potential impact upon or through the choroid plexus as a factor of pathology [113–115]. The meninges, located between the brain and the skull, consist of three layers and provide structural support and vascular pathways for CSF to gain entry to the spinal cord [109]. The layers of the meninges are comprised of the dura mater, arachnoid mater, and pia mater, with each containing distinct resident macrophages to further aid in brain protection [110, 116]. A key feature of the meninges is the glymphatic system, which includes the meningeal lymphatic vessels and is glial‐dependent, in that it requires the work of astrocyte endfeet to aid in the exchange of metabolites between the CSF and the interstitial fluid (ISF) to help eliminate products created in the CNS [117]. This is important in the context of HE, as astrocyte dysregulation has been observed in both Type A and Type C HE, and products of the CNS attempting to detoxify metabolites crossing the BBB (i.e., glutamate, ammonia, bile acids) can build up in the brain, indicating potential dysfunction of glymphatic flow. In viral infections of the CNS, the MHC‐II macrophages of the meninges appear to work synergistically with microglia to respond [116], and thus, there is growing interest in their potential role or association in HE pathogenesis.

### 5.1. Choroid Plexus

Pathology studies in the field of Type A HE have highlighted a need for investigating the role of the choroid plexus in relation to astrocyte swelling and brain edema despite a lack of breakdown of the BBB [118]. A preliminary study utilizing the Type A HE TAA model in rats had shown activated mast cells in the choroid plexus that resulted in a significant increase in cytokine production in addition to decreased IGF, FGF, and platelet‐derived growth factor [118]. Increased brain edema was observed in the treated rats alongside an increase in aquaporin 1 and 4 (AQP‐1/AQP‐4); however, inhibiting mast cell activation attenuated both of these measures. These findings indicate that the increased brain water content responsible for brain edema in Type A HE may be associated with mast cell activation resulting from ALF [118].

A more recent study investigating changes in the central ventricles and choroid plexus of mice treated with ammonium acetate modeling HE with hyperammonemia observed enlargement of the central ventricles 24 h posttreatment [119]. Histopathological assessment of the choroid plexus of mice 3 h after ammonium acetate treatment showed increased swelling, but this began to decrease after 24 h [119]. Upon observing the cell–cell junctions of the choroid plexus 3 and 24 h after treatment, capillary expansion and loosening of connective tissues had begun [119]. Intracellular density was decreased, and epithelial thinning of the choroid plexus was observed at both time points [119]. Additionally, it was differentially expressed across different regions of the choroid plexus as a result of ammonium acetate treatment [119]. Immunofluorescence results showed an increased AQP4 intensity in the apical membranes, but not the basal membranes, as seen in controls, after 3 h [119]. This was also observed in the 24‐h group. Western blot assessment of AQP4 showed a transient, but not significant, increase in AQP4 expression in the 3 and 24‐h groups; altogether indicating a potentially increased flow of CSF out of these membranes [119]. They also observed increased swelling of tissues around the cerebral cortex, validating findings in HE patients [119]. Though the concentration of ammonium acetate used in the study exceeds that observed in patients, these findings further elucidate the role of AQP4 and regulation of brain fluid levels that could contribute toward cerebral edema during HE [119]. With structural changes in the choroid plexus observed, further research investigating proinflammatory secretions during HE can resolve if the AQP4 expression levels correlate with macrophage recruitment and activation. During hydrocephalus, epithelial cells of the choroid plexus have been observed to secrete CCL2 to recruit CCR2^+^ monocytes, which induce the downstream activation of TNF‐α/NF‐κB [119]. Such recruitment may also be occurring during HE, but there have been no studies demonstrating this as of yet.

### 5.2. Meninges and Lymphatic/Glymphatic Drainage

Studies have shown that the efficacy of the glymphatic system in HE may be indirectly or indirectly related to neuroinflammation in the pathology of acute and chronic HE. It is thought that the regulation of cerebral blood flow, responsible for eliminating waste from the parenchyma of the brain and supplying the neurons of the CNS with oxygen and nutrients, is impaired in both humans and animal studies in both acute and chronic liver disease [120–124]. In humans with ALF and chronic liver disease, this impaired cerebral blood flow regulation has been associated with the swelling of astrocytes, resulting in cerebral edema [125, 126].

Notably, there is evidence of impairment of the glymphatic system in a model of Type C HE done with rats undergoing BDL surgery [127]. Glymphatic flow in these mice, as assessed by an intracisternal infusion of gadolinium and MR imaging, showed impaired penetration of the contrast agent in the olfactory bulbs and the prefrontal cortex, yet increased penetration in the hippocampus, in BDL rats [127]. This was found to be associated with decreased AQP4 immunofluorescence intensity localized to blood vessels (labeled with Isolectin), decreased AQP4 polarization to blood vessels, and decreased AQP4 vessel coverage in both the olfactory bulbs and prefrontal cortex [127]. This disruption to astrocytes and glymphatic flow was associated with decreased prefrontal cortex function in BDL rats via spatial working memory assessment [127], highlighting the role of astrocyte function at the glymphatic and meningeal level in contributing toward cognitive deficits in Type C HE. This may aid in explaining how harmful metabolites in the CSF and ISF accumulate in the brain during HE pathology when no structural deterioration of the BBB has occurred, but more studies elucidating whether the astrocyte dysregulation or reactive astrogliosis is responsible for this altered glymphatic flow rather than solely a downstream consequence is required.

In a study of the meninges and lymphatic system in a 4‐week BDL rodent model of Type C HE, rats were injected with adeno‐associated virus 8‐vascular endothelial growth factor C (AAV8‐VEGF‐C) in the cisterna magna 1‐day postsurgery and treated with archived GSE41919 to induce meningeal lymphangiogenesis neuroinflammation [128]. The cortex of the rats without AAV8‐VEGF‐C treatment showed increased microglial activation and IBA1 expression, consistent with other findings of neuroinflammation in HE, alongside increased IL‐1b, INF‐γ, and TNF‐α expression [128]. With the injection of AAV8‐VEGF‐C, the expected increase in meningeal lymphangiogenesis occurred alongside tracer dye uptake into the middle and anterior cortex [128]. This same tracer uptake was observed in the meninges, and lymph nodes were shown to be draining fluid [128]. Motor deficits assessed via Rota‐rod were attenuated in the AAV8‐VEGF‐C group [128]. Brain ammonia was attenuated, and microglial branch length was significantly increased compared to the sham group [128]. The expression of IL‐1β, INF‐γ, and TNF‐α was differentially, but significantly, attenuated in certain regions of the cortex, and IBA1 expression in all regions was significantly decreased [128]. RNA‐seq analysis also revealed the suppression of genes associated with the NF‐κB signaling pathway, Fc gamma R‐mediated phagocytosis, CNS‐associated macrophages (CAMs), cytokine–cytokine receptor interactions, and glutamatergic synapses [128]. These findings reveal that the overexpression of VEGF‐C results in increased meningeal lymphatic drainage and may be associated with a decrease in neuroinflammation to attenuate motor function deficits in the anterior and middle cortex. This study also highlights the potential role of the meninges and the lymphatic system in the pathogenesis of HE arising from chronic liver disease; however, no such studies have investigated if deficient meningeal lymphatic or glymphatic flow plays an equal or lesser role in Type A HE.

The field studying the involvement of the choroid plexus, meninges, and glymphatic system in HE pathology is quite small, and thus, more studies must be done to develop the knowledge of these regions in relation to the neuroimmune system during the pathogenesis of all types of HE to further define their role and identify future therapeutics targeted toward the treatment of HE.

## 6. Summary

HE is a serious prognostic concern for patients with liver failure and has proved to have an incredibly complex pathology, making targeted therapeutic development a difficult task. The field of HE pathogenesis research grows more robust as work aims to elucidate the full multiorgan impact and factors that ultimately lead to potentially irreversible cognitive deficits in patients, which may lower the quality of life. Neuroinflammation has proven to be a double‐edged sword with regard to neuroprotection and pathogenesis with respect to neuropsychiatric and neurologic disturbances, as outlined in this review. The main immune cell populations of the CNS, microglia, and astrocytes, have proven to play much more intricate roles in the progression of central neuroinflammation in interestingly cell‐specific manners. Pro‐inflammatory cytokine and chemokine signaling show promise in investigating both down and upstream recruitment of immune cell populations and responses during pathology. The fairly new, but promising, work investigating the choroid plexus and meninges as potential recruiters and potentiators of neuroimmune responses to CSF and blood entry of ammonia and other identified neuroimmune potentiators remains widely undiscovered and will require further studies to fully identify the full impact of these tissues. Further investigation into the interplay between the peripheral and central immune response, neurogliolymphatic system, and balance between positive and negative effects of neuroinflammation in HE may also aid in furthering global understanding of HE pathogenesis in the hopes of identifying therapeutic targets, preventative intervention strategies, and defining more robust standards of care to improve patient comfort and quality of life.

## Disclosure

The content is the responsibility of the authors alone and does not necessarily reflect the views or policies of the U.S. Department of Veterans Affairs or the United States Government.

## Conflicts of Interest

The authors declare no conflicts of interest.

## Funding

This study was funded by the National Institute of Diabetes and Digestive and Kidney Diseases (Grant DK135995).

## Data Availability

Data sharing is not applicable to this article as no datasets were generated or analyzed during the current study.
